# Machine learning-driven strategies for enhanced pediatric wheezing detection

**DOI:** 10.3389/fped.2025.1428862

**Published:** 2025-05-20

**Authors:** Hye Jeong Moon, Hyunmin Ji, Baek Seung Kim, Beom Joon Kim, Kyunghoon Kim

**Affiliations:** ^1^ Department of Pediatrics, Seoul National University College of Medicine, Seoul, Republic of Korea; ^2^ Department of Health Science and Technology, Seoul National University, Seoul, Republic of Korea; ^3^ Department of Pediatrics, Seoul National University Bundang Hospital, Seongnam, Republic of Korea; ^4^ Department of Pediatrics, College of Medicine, The Catholic University of Korea, Seoul, Republic of Korea

**Keywords:** wheezing detection, machine learning, pediatric, AI strategies, wheezing

## Abstract

**Background:**

Auscultation is a critical diagnostic feature of lung diseases, but it is subjective and challenging to measure accurately. To overcome these limitations, artificial intelligence models have been developed.

**Methods:**

In this prospective study, we aimed to compare respiratory sound feature extraction methods to develop an optimal machine learning model for detecting wheezing in children. Pediatric pulmonologists recorded and verified 103 instances of wheezing and 184 other respiratory sounds in 76 children. Various methods were used for sound feature extraction, and dimensions were reduced using t-distributed Stochastic Neighbor Embedding (t-SNE). The performance of models in wheezing detection was evaluated using a kernel support vector machine (SVM).

**Results:**

The duration of recordings in the wheezing and non-wheezing groups were 89.36 ± 39.51 ms and 63.09 ± 27.79 ms, respectively. The Mel-spectrogram, Mel-frequency Cepstral Coefficient (MFCC), and spectral contrast achieved the best expression of respiratory sounds and showed good performance in cluster classification. The SVM model using spectral contrast exhibited the best performance, with an accuracy, precision, recall, and F-1 score of 0.897, 0.800, 0.952, and 0.869, respectively.

**Conclusion:**

Mel-spectrograms, MFCC, and spectral contrast are effective for characterizing respiratory sounds in children. A machine learning model using spectral contrast demonstrated high detection performance, indicating its potential utility in ensuring accurate diagnosis of pediatric respiratory diseases.

## Introduction

Wheezing is defined as the rapid movement of air through narrowed airways caused by bronchial asthma, allergic reactions, or respiratory infections (1). Wheezing is characterized by sinusoidal oscillations of 100–1,000 Hz and can occur during both inhalation and exhalation. Wheezing is an important symptom in the diagnosis of various diseases. For example, in asthma and chronic obstructive pulmonary disease, wheezing can be heard in any part of the chest due to airway narrowing in the anterior lung fields. However, local bronchial obstruction due to foreign bodies, mucus, or narrowing due to tumors may cause wheezing predominantly in specific areas (2).

In general practice, lung diseases are diagnosed by clinicians following an examination of the patient's chief complaint, medical history, physical examination, and auscultatory findings. Distinguishing between wheezing and non-wheezing during auscultatory examination, which is key in the diagnosis of many lung diseases, requires years of training and experience, and is open to individual subjectivity. This makes objective assessment difficult. Furthermore, in high risk patients requiring isolation, direct physical examination is limited, which limits auscultatory assessment (3–5).

To overcome these limitations, researchers have used artificial intelligence (AI) to distinguish between normal and abnormal auscultatory sounds, with some studies indicating better performance than human doctors (6, 7). In particular, the International Conference on Biomedical and Health Informatics open dataset has been extensively studied for auscultatory sound classification (8–10). In these studies, preprocessing methods were used to extract audio features for AI training. These features included the Mel-spectrogram, log-Mel-spectrogram, and Mel-frequency cepstral coefficient (MFCC), which are known to represent audio data (11, 12).

This study was conducted to determine the most effective tool for the extraction of features from wheezing sounds. Feature extraction was performed using various sound classification tools. To observe the differences in performance, we used a kernel support vector machine (SVM), a type of machine learning model, to classify wheezing (13). In addition, we compared the dimensionality of different features extracted from audio data by t-stochastic neighbor embedding (t-SNE) by reducing and visualizing them, and analyzed which features could be used to learn and represent breathing sound data well (14). The overall aim of this project was to determine the existing techniques that are effective in distinguishing between breathing sounds.

## Methods

### Study design and data collection

We conducted a prospective study of pediatric patients who visited the pediatric department of a university hospital in Korea between August 2019 and January 2020. All records were obtained from patients who voluntarily agreed to have their breath sounds recorded. All breath sounds were recorded in children visiting the outpatient department by a pediatric respiratory specialist using an electronic stethoscope (Jabes, GST Technology, Seoul, Korea).

The recorded auscultatory sounds were categorized as wheezing or non-wheezing according to the pediatric physician's diagnosis. Two auscultation cycles were recorded for each patient, with one in the anterior lung fields and the other in the posterior lung fields. Two breath sound recordings were obtained during each cycle, resulting in a total of four recordings per participant. This standardized approach ensured consistent data collection across all participants. To validate the classification, two board-certified pediatric respiratory specialists independently reviewed all recorded breath sounds under blinded conditions. Their evaluations were based on standard clinical auscultation criteria for wheezing. A recording was flagged and included in the final dataset only if both reviewers independently agreed with the original classification. This conservative inclusion criterion was applied to ensure the reliability and consistency of the labeled data. Data on sex, age, and auscultation site were also collected.

### Feature extraction

In this study, the following feature extraction methods were used to extract 48 kH breath sound data:

(1) Mel-spectrogram: This is a popular feature extraction method which is used to analyze data with frequency characteristics that change over time. A Mel-spectrogram is output after the audio data have been subjected to a Fast Fourier Transform and passed through a Mel filter bank.
(2) Log Mel-spectrogram: This method takes the logarithm of the Mel-spectrogram and converts it to a frequency similar to that heard by humans.
(3) MFCC: This feature extraction method performs a Discrete Cosine Transform (DCT) operation on a Mel-spectrogram. It is primarily used for human speech data, and requires less computation than the Mel-spectrogram (15).
(4) MFCC-delta: This is a method of stacking the MFCC and deltas (first differences) and delta-deltas (second differences) for the MFCC around the frequenc*y* axis, representing noisy data (16).
(5) Chroma Short-Time Fourier Transform (STFT): This is a feature for the representation of a 12 tone scale, often used in the analysis of music data (17).
(6) Chroma Constant-Q Transform (CQT): This application uses CQT instead of SFTF in the chroma. It considers the geometric split between different frequency bands and contains additional high-frequency information (18).
(7) Spectral contrast: This method is based on differences in spectral contrast, where higher frequencies are contrasted with lower frequencies to create a more pronounced difference (19).
(8) Tonnetz: This method incorporates the tonnet theory discovered by Euler and is effective in uncovering hidden relationships and patterns (20).

Mel-spectrogram, MFCC, and spectral contrast features were extracted using commonly adopted default parameters provided by standard audio processing libraries. For example, MFCCs were computed using 13 coefficients derived from the log-Mel spectrogram. These parameter settings, commonly adopted in respiratory sound analysis, were chosen to ensure consistent and reliable feature extraction across recordings.

### Evaluation of the AI algorithm

**Figure 1:**
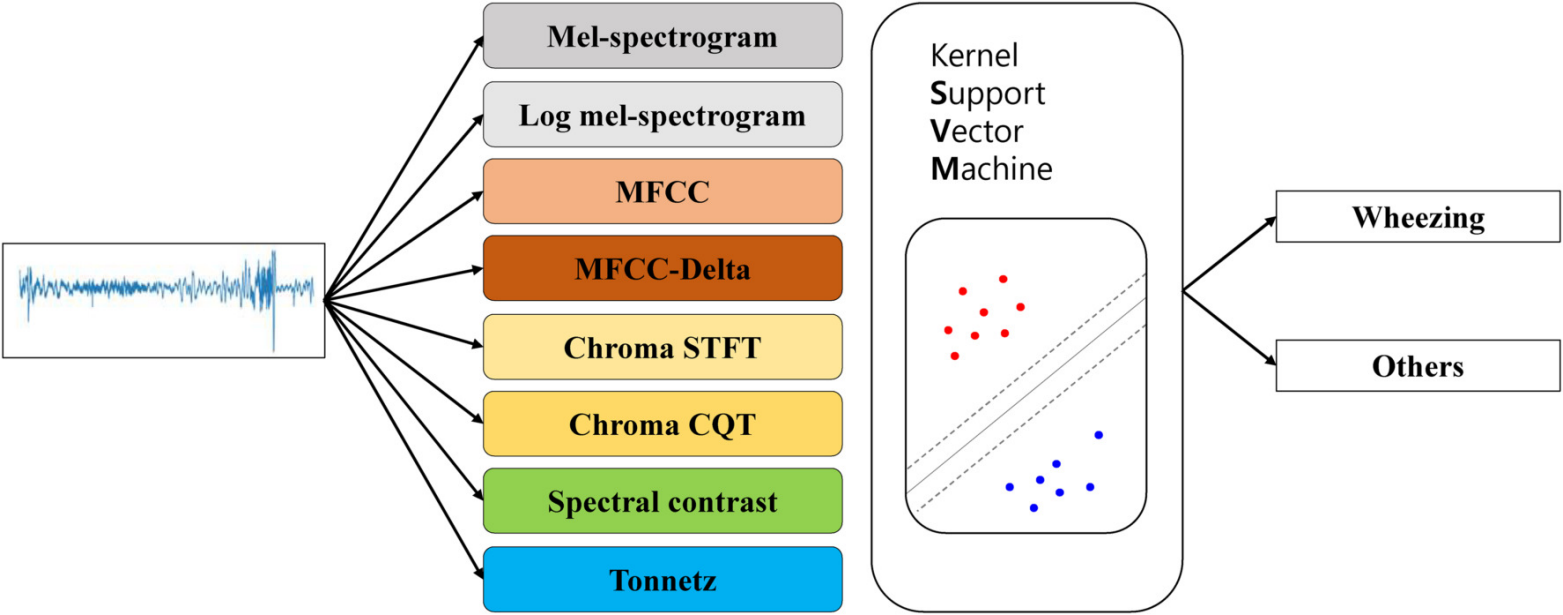
Flowchart showing the use of t-SNE to visualize lung sounds. MFCC, mel-frequency cepstral coefficient; STFT, short-time fourier transform; CQT, constant-Q transform; t-SNE, t-stochastic neighbor embedding.

The kernel SVM was used to classify wheezing and non-wheezing sounds for each feature obtained from the breath sound data (Figure 1). To compare the distribution of features, t-SNE was used solely for visualization purposes by reducing the dimensionality to a two-dimensional coordinate space.

An SVM is a supervised learning algorithm that aims to classify two categories by finding the optimal decision boundary between them. Kernel SVM was chosen based on its proven performance in small- to medium-sized datasets and its successful application in prior respiratory sound classification studies. Kernel SVMs apply a kernel trick to SVMs that allows them to classify multidimensional data linearly (14). Both linear and radial basis function (RBF) kernels were tested across feature types. Kernel selection was based on validation performance, with RBF applied to MFCC and chroma-based features, and linear kernels retained for others such as Mel-spectrogram and spectral contrast. Additionally, the hyperparameter search ranges used in the grid search were selected based on standard practices commonly adopted in prior respiratory sound classification studies. In the present study, we performed 5-fold cross-validation and grid search on the training data (80% of the total data) to explore the optimal hyperparameters of the kernel SVM for training and compared the final results with the test data (20% of the total data) (Tables 1, 2).

**Table 1:** Selected hyperparameters with grid search.

Feature	Type	Kernel	Gamma
Mel-spectrogram	Linear	0.1	1,000
Log Mel-spectrogram	Linear	0.1	1,000
MFCC	RBF	0.1	100
MFCC-Delta	RBF	0.1	1,000
Chroma STFT	RBF	1	0.1
Chroma CQT	RBF	1,000	0.01
Spectral contrast	Linear	0.1	1,000
Tonnetz	Linear	0.1	1,000

MFCC, mel-frequency Cepstral Coefficient; STFT, short-time fourier transform; CQT, constant-Q transform; RBF, radial basis function.

**Table 2:** Performance of the different models in discriminating other respiratory sounds from wheezing use kernel support vector machine.

Feature	Accuracy	AUC	Precision	Recall	F1-score
Mel-spectrogram	0.862	0.871	0.760	0.905	0.826
Log Mel-spectrogram	0.845	0.868	0.714	0.952	0.816
MFCC	0.863	0.882	0.741	0.952	0.833
MFCC-Delta	0.810	0.799	0.727	0.761	0.744
Chroma STFT	0.724	0.722	0.600	0.714	0.652
Chroma CQT	0.689	0.654	0.579	0.524	0.550
Spectral contrast	0.897	0.909	0.800	0.952	0.869
Tonnetz	0.672	0.651	0.545	0.571	0.558

MFCC, mel-frequency cepstral coefficient; STFT, short-time fourier transform; CQT, constant-Q transform.

t-SNE is a machine learning algorithm designed to reduce the dimensionality of high-dimensional data to facilitate the visualization of vectors. It computes probability values for each dimension based on the SNE framework to effectively preserve the pairwise distances between vectors during the dimension reduction process. Specifically, it quantifies the likelihood that data points are chosen as neighbors in the original high-dimensional setting.

Similarly, the dimension reduced post-analysis, t-SNE defines the probability that pairs of data points are selected as neighbors in the lower-dimensional space.

To ensure symmetry in these relationships, t-SNE modifies the probabilities to consider the mutual probability of each pair equally. This symmetry, maintained in both directions between two points, ensures that the relationships are consistent. This approach helps in maintaining the local structure of the data during the dimensionality reduction process.

We defined Kullback-Leibler divergence (KL) divergence as a cost function that measures the similarity of corresponding distributions. This divergence quantifies how one probability distribution diverges from a second, expected probability distribution (21).

The training process uses gradient descent to minimize the cost function and enhance the model's accuracy in distinguishing data distributions.

In short, t-SNE learns the equivalent Euclidean distance for both pre- and post-decreasing dimensionality, albeit with fewer dimensions.

**Figure 2:**
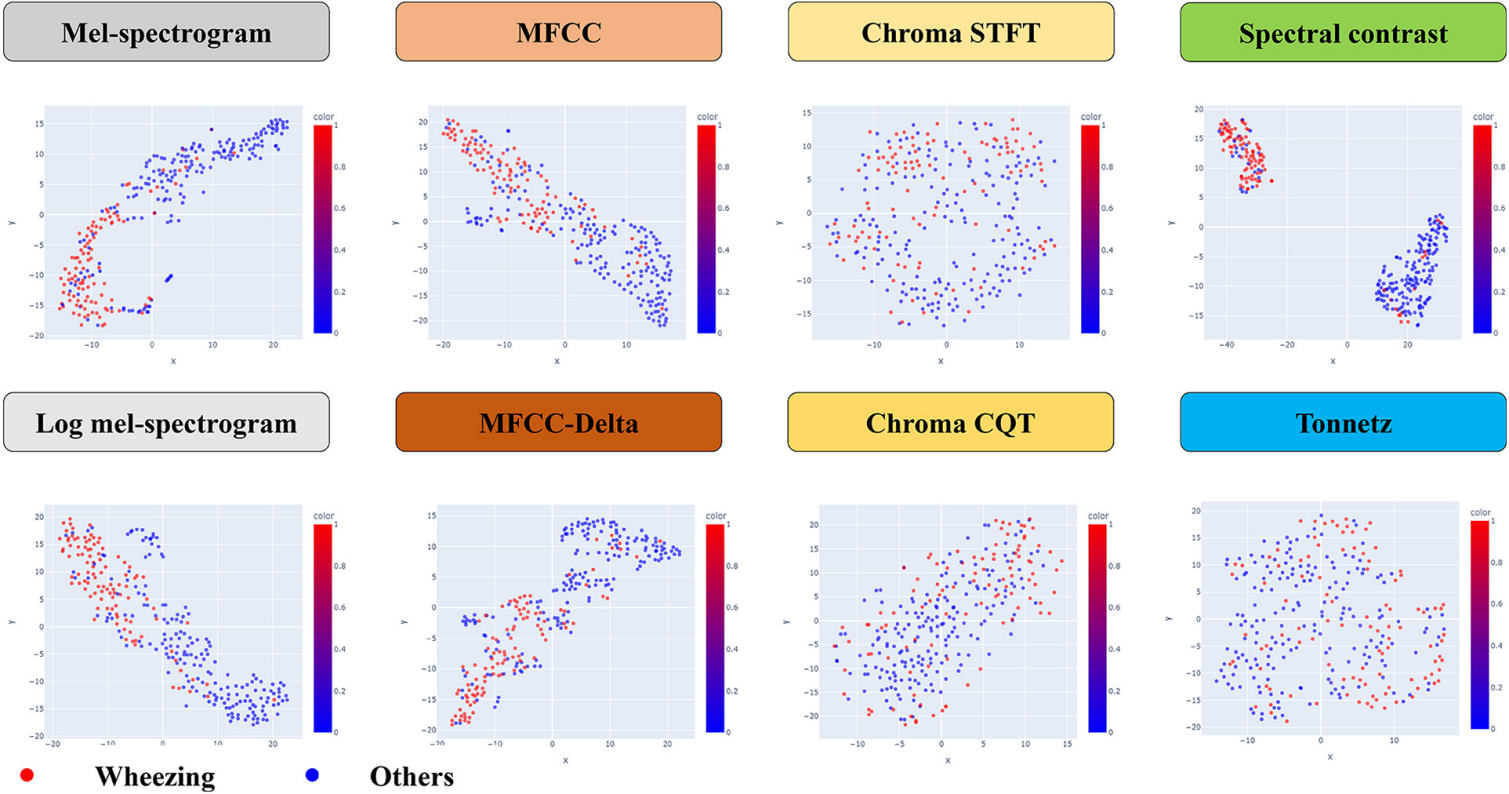
The structure of a kernel support vector machine model. MFCC, mel-frequency cepstral coefficient; STFT, short-time fourier transform; CQT, constant-Q transform.

The dimensionality was reduced to a two-dimensional coordinate plane for the values of each feature on the x- and y-axes, and wheezing and non-wheezing participants were visualized in two separate classes (Figure 2). This study was conducted using Python software version 3.6.5 (Python Software Foundation, 9450 SW Gemini Dr., ECM# 90772, Beaverton, OR 97008, USA) and the Librosa package was used for each feature extraction. The scikit-learn package was used to model t-SNE and SVM.

### Statistical analysis

Statistical analysis was conducted using the data extracted for each feature, utilizing accuracy, area under the curve (AUC), precision, recall, and F1-scores.

### Ethics statement

This study was approved by the Institutional Review Board (IRB) of the Catholic University of Korea (IRB approval no. PC19OESI0045). Written informed consent was obtained from at least one legal guardian for all participants. For children 7 years of age and older, assent of child was also obtained. All methods were performed in accordance with relevant guidelines and regulations.

## Results

A total of 76 patients were included in the study, and 103 wheeze sounds and 184 non-wheeze sounds were collected. Based on these data, the characteristics of the auscultatory sounds were summarized according to sex, age, and duration of breath sounds (Table 3). The median age of the patients with wheezing was 4 years (2–8 years), while that in those without wheezing was 3 years (1–5 years). We found that the duration times of the wheezing participants were 89.36 ± 39.51 ms and those of the non-wheezing participants were 63.09 ± 27.79 ms.

**Table 3:** Characteristics of respiratory sounds collected in the study.

Variable	Wheezing	Others	*P* value
	(*n* = 103)	(*n* = 184)	
Demographic data of the included patients			
Male sex, *n* (%)	67 (65.0)	113 (61.4)	0.541
Age (years)	4 (2–8)	3 (1–5)	<0.001
Duration of sound (ms)	89.36 ± 39.51	63.09 ± 27.79	<0.001

Continuous variables are expressed as mean ± standard deviation or median (interquartile range).

The kernel SVM was used to classify wheezing and non-wheezing sounds for each feature obtained from the breath sound data (Figure 2). The audio data were extracted using the Mel-spectrogram, log-Mel-spectrogram, MFCC, MFCC-delta, chroma STFT, chroma CQT, spectral contrast, and tonnet feature extraction methods. To determine the best-performing SVM, the size of each region was determined based on the kernel and gamma values using a grid search. This type distinguishes this area into two types of lines: a linear type for linear separation and a Radial Basis Function as a curve that follows a normal distribution shape to divide each area. These are summarized based on the hyperparameter tuning results for each model (Table 1).

In the statistical analysis, the AUC and F1-scores are metrics that can be indicative of data imbalance and were found to be effective in this study. The Mel-spectrogram, MFCC, and spectral contrast proved to be the most suitable for classifying breath sounds, demonstrating the clearest clustering in distinguishing between wheezing and non-wheezing sounds.

In particular, spectral contrast achieved an AUC of 0.909 and an F1-score of 0.869, indicating the highest classification performance (Table 2).

## Discussion

In this study, we investigated whether any of the existing machine learning techniques can effectively distinguish between lung disease patients with and without wheezing by recognizing specific diagnostic patterns from breathing data. A total of 76 patients were included, and 103 wheeze sounds and 184 non-wheeze sounds were analyzed. This class distribution resulted from the diagnostic classification process, as each participant contributed the same number of recordings. Given the relatively mild class imbalance, we did not apply resampling or class-weighting. Instead, model performance was assessed using F1-score, recall, and AUC, which are well-suited to imbalanced classification scenarios. Based on the breathing voice data, the Mel-spectrogram, MFCC and spectral contrast were found to be the most suitable for classifying breathing sounds and distinguishing between wheezing and non-wheezing sounds with clear clustering. Among the various techniques analyzed, spectral contrast demonstrated the most effective classification performance in distinguishing wheezing in children. This suggests that machine learning models using spectral contrast may be used to accurately diagnose respiratory diseases in children. By systematically comparing these methods, our work provides a useful foundation for future efforts to optimize machine learning models for pediatric wheeze classification.

Meanwhile, differentiation between wheezing and non-wheezing requires extensive medical expertise, impeding objective evaluations and restricting the use of auscultatory assessments in isolated, high risk populations. To address these issues, researchers have turned to deep learning methods to differentiate between normal and abnormal auscultatory sounds. Recently, several techniques have been proposed to improve the identification of lung sounds using deep learning. Efforts have been made to categorize breath noise by applying traditional deep learning neural networks (CNNs) (22, 23). In a recent study, a CNN model was used to distinguish wheezing sounds (5). Further, machine learning has been used to classify abnormal respiratory sounds into subclasses. Different architectures were shown to effectively differentiate between wheeze, rhonchi, and crackles. However, the authors opted for CNNs over SVMs for the connection between the feature extractor and classifier as CNNs were found to yield superior results in both image classification and traditional classification tasks. The researchers opted for a CNN as the classifier and utilized InceptionV3, DenseNet201, ResNet50, ResNet101, VGG16, and VGG19 as feature extractors. The study's findings revealed that VGG16 yielded the most favorable results by achieving an AUC of 0.93 and accuracy of 86.5%, validating its competence in identifying anomalous lung sounds and classifying crackles, wheezes, and rhonchi (24).

Previous studies have analyzed feature extractors and classifiers for integrating breath sounds into machine learning with a focus on determining the most effective models. However, which method is superior for distinguishing wheezing sounds remains unknown. Numerous techniques are currently available to extract features from breath sounds; however, none have been identified as being particularly effective in distinguishing wheezing. The raw audio data functionally represents the pitch as sound pressure over time. In recent deep learning and machine learning methods, features are extracted from raw data rather than from raw audio data. Feature extraction techniques enable frequency representation by decomposing the time data into frequency components using a Fast Fourier Transform. This conversion process reveals which frequencies are strong or weak in the audio signal and how deep learning or machine learning can better learn from audio data. Furthermore, there are several techniques for extracting features from audio, and the manner in which these features are extracted is critical for accurately representing audio. These features have a horizontal (time) axis, vertical (frequency) axis, and one channel, creating an image-like data structure.

We found that the spectral contrast performed best as a feature extractor for wheezing. This performance remains meaningful even when compared to studies using external datasets. For instance, a recent ICBHI-based study reported an F1-score of 0.69 and recall of 74% using a MobileNet-based multi-task learning model (25). Despite differences in datasets and model complexity, our SVM-based approach achieved comparable or superior results (F1-score: 0.869; recall: 95.2%), suggesting the potential utility of spectral contrast features in interpretable and efficient models for pediatric wheeze detection. Furthermore, we did not limit ourselves to the extraction and classification of low-frequency wheezing sound data. Instead, we employed t-SNE to reduce dimensionality and trained machine learning models using this approach. As previously noted, this study differs from others in that the data were classified using t-SNE based on multiple features and then reduced to a two-dimensional coordinate plane, allowing for the visualization of wheezing and non-wheezing. However, this study had some limitations. It was conducted on a single-center basis, and the limited sample size during the collection of low data made assessment of accuracy challenging. For these reasons, we were restricted to evaluating the effectiveness of the hyperparameters solely using the AUC and F1-score metrics. Moreover, because we trained the machine learning model to differentiate only between wheezing and non-wheezing respiratory sounds, it remains unclear whether spectral contrast provides a similarly outstanding performance when distinguishing other respiratory sound types. Validating large-scale prospective studies is essential for future research, although utilizing spectral contrast may improve the performance of AI in distinguishing respiratory sound characteristics. Building on our findings, future studies could incorporate diverse and independent datasets, such as the ICBHI wheezing dataset or HF_Lungs dataset, to further evaluate the model's performance and strengthen its robustness across varied clinical conditions and populations. Additionally, while we prioritized machine learning models for their interpretability and computational efficiency, comparing our approach with state-of-the-art deep learning methods, such as ResNet18 or Audio Spectrogram Transformers, could provide further understanding of model optimization strategies.

## Conclusion

Accurate diagnosis of wheezing in children is essential, as wheezing is a key clinical sign of respiratory diseases such as asthma. This study confirmed that the Mel-spectrogram, MFCC, and spectral contrast exhibited the best performance in characterizing respiratory sounds. Overall, we found that machine learning trained on spectral contrast demonstrated superior performance in detecting wheezing sounds compared to other feature extraction methods in pediatric cases. These findings suggest that high-performance machine learning models utilizing spectral contrast may support more accurate analysis of pediatric respiratory sounds and contribute to improved precision in detecting abnormal breathing patterns.

## Data Availability

The original contributions presented in the study are included in the article/Supplementary Material, further inquiries can be directed to the corresponding author.
